# Identification of a Wheat-*Psathyrostachys huashanica* 7Ns Ditelosomic Addition Line Conferring Early Maturation by Cytological Analysis and Newly Developed Molecular and FISH Markers

**DOI:** 10.3389/fpls.2021.784001

**Published:** 2021-12-09

**Authors:** Binwen Tan, Lei Zhao, Lingyu Li, Hao Zhang, Wei Zhu, Lili Xu, Yi Wang, Jian Zeng, Xing Fan, Lina Sha, Dandan Wu, Yiran Cheng, Haiqin Zhang, Guoyue Chen, Yonghong Zhou, Houyang Kang

**Affiliations:** ^1^Triticeae Research Institute, Sichuan Agricultural University, Chengdu, China; ^2^State Key Laboratory of Crop Gene Exploration and Utilization in Southwest China, Sichuan Agricultural University, Chengdu, China; ^3^College of Resources, Sichuan Agricultural University, Chengdu, China; ^4^College of Grassland Science and Technology, Sichuan Agricultural University, Chengdu, China

**Keywords:** *Psathyrostachys huashanica*, ditelosomic addition line, early maturation, SLAF-seq, molecular marker, FISH probes

## Abstract

Early maturation is an important objective in wheat breeding programs that could facilitate multiple-cropping systems, decrease disaster- and disease-related losses, ensure stable wheat production, and increase economic benefits. Exploitation of novel germplasm from wild relatives of wheat is an effective means of breeding for early maturity. *Psathyrostachys huashanica* Keng f. ex P. C. KUO (2*n*=2*x*=14, NsNs) is a promising source of useful genes for wheat genetic improvement. In this study, we characterized a novel wheat-*P. huashanica* line, DT23, derived from distant hybridization between common wheat and *P. huashanica*. Fluorescence *in situ* hybridization (FISH) and sequential genomic *in situ* hybridization (GISH) analyses indicated that DT23 is a stable wheat-*P. huashanica* ditelosomic addition line. FISH painting and PCR-based landmark unique gene markers analyses further revealed that DT23 is a wheat-*P. huashanica* 7Ns ditelosomic addition line. Observation of spike differentiation and the growth period revealed that DT23 exhibited earlier maturation than the wheat parents. This is the first report of new earliness *per se* (*Eps*) gene(s) probably associated with a group 7 chromosome of *P. huashanica*. Based on specific locus-amplified fragment sequencing technology, 45 new specific molecular markers and 19 specific FISH probes were developed for the *P. huashanica* 7Ns chromosome. Marker validation analyses revealed that two specific markers distinguished the Ns genome chromosomes of *P. huashanica* and the chromosomes of other wheat-related species. These newly developed FISH probes specifically detected Ns genome chromosomes of *P. huashanica* in the wheat background. The DT23 line will be useful for breeding early maturing wheat. The specific markers and FISH probes developed in this study can be used to detect and trace *P. huashanica* chromosomes and chromosomal segments carrying elite genes in diverse materials.

## Introduction

Early maturation is an important target in wheat breeding programs. In recent years, breeding efforts have targeted increase in wheat yield potential to meet the growing food demand resulting from global population explosion, climate change, and reduction in arable land area (Mondal et al., 2013; Chen et al., 2016). Certain adverse factors, such as Fusarium head blight, powdery mildew, and rust infection, high temperature, frost damage, excessive rainfall, and preharvest sprouting, are liable to occur during the mid to late wheat-growing season and comprise the primary causes of reduction in wheat yield and quality (Iqbal et al., 2006; Mahdiyeh and Bahram, 2012; Sheehan and Bentley, 2020). Exploitation of new early maturation genes and the breeding of early maturing wheat cultivars are strategies proven to be effective against these emerging threats to agricultural production and are suitable for a double-cropping management system, in which earlier sowing of the following crop improves the probability of its success (Hunger et al., 2014; Su et al., 2018). Such an approach promotes effective utilization of the limited arable land and enhances grain yield.

The wheat heading stage is jointly controlled by vernalization (*Vrn*), photoperiod (*Ppd*), and earliness *per se* (*Eps*) genes (Shi et al., 2019). Given their involvement in essential growth and development processes, these genes are crucial in determining the length of the wheat growth period (Zhao et al., 1989). The *Ppd* and *Vrn* genes show significant interactions with the environment, whereas *Eps* genes participate at various stages of wheat growth and function independently (Ochagavía et al., 2018; Shi et al., 2019). To date, a number of *Eps* quantitative trait loci (QTLs) have been mapped on different wheat and barley chromosomes (Laurie et al., 1995; Kamran et al., 2014; Lombardo et al., 2019). Only a small number of *Eps* genes originating from wheat relatives have been exploited, such as *LUX* and *ELF3* derived from *Triticum monococcum* (Gawroński and Schnurbusch, 2012; Gawroński et al., 2014; Alvarez et al., 2016), and *HvCEN* derived from *Hordeum vulgare*, respectively (Comadran et al., 2012). Therefore, the discovery and identification of additional sources of early maturation in wheat-related germplasm are an important and long-term objective for breeding early maturing wheat cultivars.

*Psathyrostachys huashanica* Keng f. ex P. C. KUO (2*n*=2*x*=14, NsNs) is a diploid, perennial, outcrossing graminaceous species restricted to the Huashan section of the Qinling Mountains, Shaanxi Province, China (Baden, 1991). As a wild relative of common wheat, *P. huashanica* is favored by many wheat breeders because it possesses numerous agronomically beneficial traits, such as early maturity, dwarf stature, disease resistance (to scab, stripe rust, powdery mildew, and take-all), and tolerance to abiotic factors (cold, drought, salinity, and infertile soil; Chen et al., 1991; Kang et al., 2016). To transfer these desirable genes from *P. huashanica* to wheat, distant hybridization between common wheat and *P. huashanica* has been performed since the 1980s, from which a heptaploid hybrid H8911 (2*n*=7*x*=49, AABBDNs) was successfully obtained by embryo culture (Chen et al., 1991). Subsequently, derivative lines with *P. huashanica* chromosome(s) incorporated into the common wheat background were generated, such as an amphiploid line (PHW-SA, 2*n*=8*x*=56, AABBDDNsNs; Kang et al., 2009), and chromosome addition and substitution lines (Zhao et al., 2004, 2010; Kishii et al., 2010). In the last decade, a series of wheat-*P. huashanica* derivative lines were developed and identified using molecular cytological methods, including wheat-*P. huashanica* 1Ns-7Ns disomic addition lines (Du et al., 2013a,b,c, 2014a,b,c,d), 1Ns(1D), 2Ns(2D), 3Ns(3D), and 5Ns(5D) disomic substitution lines (Li et al., 2019 2021a Bai et al., 2020; Qu et al., 2021), and several translocation lines (Kang et al., 2016; Li et al., 2020a; Liu et al., 2021). These progeny lines outperformed their wheat parents with regard to disease resistance and agronomic traits, demonstrating that *P. huashanica* is a superior wild relative useful to wheat breeding programs. However, the transfer of elite *Eps* genes carried by *P. huashanica* to common wheat has been neglected to date.

Our research team has performed wide hybridization between common wheat and *P. huashanica* since 2004 and successfully obtained F_1_ hybrids without adoption of an embryo rescue technique (Kang et al., 2008). Subsequently, wheat-*P. huashanica* derivative lines were generated by backcrossing and selfing (Wang et al., 2011). In the present study, we identified a novel wheat-*P. huashanica* 7Ns ditelosomic addition line, DT23, from offspring lines that exhibited earlier maturation than the wheat parents by fluorescence *in situ* hybridization (FISH), genomic *in situ* hybridization (GISH), FISH painting, and PCR-based landmark unique gene (PLUG) marker analyses. Furthermore, we developed and validated new specific molecular markers and specific FISH probes, based on specific locus amplified fragment sequencing (SLAF-seq) technology, to efficiently trace *P. huashanica* 7Ns chromatin in wheat breeding programs and identify chromosomes of *P. huashanica* and other wheat-related species.

## Materials and Methods

### Plant Materials

Wheat (*Triticum aestivum* L.) “Chinese Spring” (CS; 2*n*=6*x*=42, AABBDD) is a Sichuan white-grained cultivar. Chinese Spring *ph2b* (CS*ph2b*), a chemically induced mutant, was produced by a terminal segment deletion or point mutation of *ph2* gene on the short arm of CS chromosome 3D (Wall et al., 1971; Sears, 1982). *Psathyrostachys huashanica* accession ZY3156 (2*n*=2*x*=14, NsNs) was collected from the Huashan Mountains, Shaanxi Province, China, by Profs. C. Yen and J.L. Yang of Sichuan Agricultural University. We first crossed CS*ph2b* with *P. huashanica*, and thereafter, the F_1_ hybrids were crossed with CS, as the male parent, to obtain the BC_1_F_1_ generation (Kang et al., 2008). Subsequently, a wheat-*P. huashanica* 7Ns ditelosomic addition line, designated DT23, was developed through three consecutive cycles of self-pollination. Finally, molecular markers and FISH probes were validated in other wheat-related species listed in Table 1. All plant materials used are preserved in the herbarium of the Triticeae Research Institute, Sichuan Agricultural University, China.

**Table 1:** List of 17 wheat-related species used in this study.

Materials	Accession	Chromosome numbers (2*n*)	Ploidy	Genome
*Triticum urartu*	PI142824	14	2×	A^u^
*Aegilops speltoides*	PI560527	14	2×	B^sp^
*Aegilops tauschii*	PI508264	14	2×	D
*Psathyrostachys huashanica*	ZY3156	14	2×	Ns
*Psathyrostachys juncea*	PI314082	14	2×	Ns
*Secale cereale*	QL	14	2×	R
*Hordeum vulgare*	ZY11001	14	2×	H
*Agropyron cristatum*	PI499389	14	2×	P
*Dasypyrum villosum*	PI470279	14	2×	V
*Thinopyrum bessarabicum*	W6-10232	14	2×	E^b^
*Thinopyrum elongatum*	PI531718	14	2×	E^e^
*Pseudoroegneria libanotica*	PI228391	14	2×	St
*Leymus racemosus*	ZY07023	28	4×	NsXm
*Leymus secalinus*	ZY09002	28	4×	NsXm
*Leymus coreanus*	PI531578	28	4×	NsXm
*Leymus multicaulis*	PI440326	28	4×	NsXm
*Leymus arenarius*	PI294582	56	8×	NsXm
*Leymus cinereus*	PI232252	56	8×	NsXm

### FISH and Sequential GISH Analyses

Actively growing root tips from seeds germinated at 22°C in a constant temperature incubator were treated with nitrous oxide gas for 2.5h and 90% glacial acetic acid for at least 10min and then digested with pectinase and cellulase (Komuro et al., 2013). Mitotic chromosome spreads from root tip cells were prepared and observed for the FISH and sequential GISH analyses using a previously described method (Han et al., 2006), with minor modifications. For the FISH experiment, a pair of fluorescent-modified probes comprising oligo-pSc119.2 (6-FAM-5′) and oligo-pTa535 (TAMRA-5′), synthesized by Sangon Biotech (Chengdu, China; Tang et al., 2014), were used to distinguish each wheat chromosome of DT23. The FISH procedure was performed as described by Han et al. (2006) and Gong et al. (2019). The chromosomes were counterstained with 4,6-diamino-2-phenylindole solution (Vector Laboratories, Burlingame, CA, United States). Mitotic chromosome number counts and fluorescent signals were visualized and captured using a fluorescence microscope (Olympus BX63) equipped with a Photometric SenSys DP-70 CCD camera (Olympus, Tokyo, Japan). The images were optimized for contrast and brightness using Adobe Photoshop software.

The slides photographed were eluted sequentially in 75% alcohol for 10min, 2× SSC in boiling water for 5min, 75% alcohol for 20min, and 100% alcohol for 20min, then exposed to bright light for 48h, and finally were prepared for sequential GISH. In the sequential GISH analysis, total genomic DNA of *P. huashanica* and *T. aestivum* “J-11” was extracted from fresh leaves using the improved cetyltrimethylammonium bromide method (Cota-Sánchez et al., 2006). Genomic DNA of the former species was labeled with Texas Red-12-dUTP (Red) using the nick translation method (Thermo Fisher Scientific, Eugene, OR, United States) and served as a probe, whereas genomic DNA of the latter cultivar was used as blocking DNA with a probe:block ratio of 1:150. The sequential GISH protocol was performed in accordance with that of Han et al. (2006) and Gong et al. (2019). Detection and visualization of GISH signals were performed as described above.

### FISH Painting Analysis

Seven bulked oligonucleotide-based FISH probes (Synt1 to Synt7) derived from single-copy sequences on chromosomes 1 to 7 of barley and corresponding to each of the seven Triticeae linkage groups (Li et al., 2021b), which were kindly provided by Dr. ZJ Yang, University of Electronic Science and Technology of China, Chengdu, China, were used to determine the homoeologous group relationships of the introduced *P. huashanica* chromosomes in line DT23. FISH painting with the bulked oligo probes was performed as described previously by Han et al. (2015) and Bi et al. (2020). After the bulked oligo-based FISH, sequential FISH and GISH were similarly conducted as described above.

### PLUG Marker Analysis

Primer pairs for 135 PLUG markers distributed evenly among the seven wheat homoeologous groups (Ishikawa et al., 2009) were employed to determine the homoeologous group relationships of the added *P. huashanica* chromosomes in line DT23. CS and CS*ph2b* were used as negative controls, whereas *P. huashanica* was used as a positive control. PCR amplification was performed as described previously by Yang et al. (2020) with slight modifications.

### Observation of Spike Differentiation and Growth Period

Plants of CS, CS*ph2b*, and DT23 were grown in the field in Wenjiang district, Sichuan Province, China, during the 2020–2021 growing season. The field layout consisted of plots 1.5m in width with 30cm rows and a sowing density of 15 plants per row. In accordance with the criteria described by Cui et al. (2008), wheat spike differentiation was categorized into eight stages, comprising apex elongation, single-ridge, double-ridge, glume primordia differentiation, floret primordia differentiation, stamen and pistil differentiation, anther separation, and tetrad stages. During the three-leaf stage to the heading stage, spike differentiation was observed with a stereomicroscope (ZEISS SteREO Discovery.V20) at seven-day intervals. Ten randomly selected individuals from each material were observed at each time point. In addition, the timing of stages in the growth period of each material was assessed, comprising the seedling, three-leaf, tillering, jointing, booting, heading, flowering, and maturity stages (Guo and Fan, 2011).

### Evaluation of Agronomic Performance

Morphological traits of DT23 and its wheat parents were evaluated in the field in Wenjiang district, Sichuan Province, China, with three replications in the 2020–2021 growing season. At the physiological maturity stage, 20 randomly selected plants of each line were harvested to evaluate their morphological traits, comprising plant height, tiller number, spike length, number of spikelets per spike, number of kernels per spike, and 1,000-grain weight. The IBM SPSS Statistics 24.0 software package was used for statistical analysis of the data.

### Molecular Marker Development

Genomic DNA of CS*ph2b*, *P. huashanica*, and DT23 was sequenced using the SLAF-seq technique (Biomarker, Beijing, China). Genomic DNA digestion, PCR fragment amplification, fragment selection, and SLAF-seq library construction were conducted as previously described by Sun et al. (2013) with slight modifications. Amplicons with appropriate sizes of 464–494bp were excised and diluted for sequencing using an Illumina HiSeq 2,500 platform (Illumina, Inc., San Diego, CA, United States). The SLAFs were identified, filtered, and clustered following the methods described by Chen et al. (2013). *Psathyrostachys huashanica* 7Ns chromosome-specific sequences were generated as follows. The high-quality DT23 sequences were first compared with the CS reference genome sequence^1^ using the Burrows-Wheeler Alignment software. The sequences with 0% similarity to CS were selected. These sequences were then compared with the CS*ph2b* sequences acquired using SLAF-seq in this study, and the sequences with identities less than 23% were selected. Finally, the retained DT23 sequences were compared with the sequences of *P. huashanica* and the sequences with identities greater than 90% were selected, which were regarded to be the *P. huashanica* 7Ns chromosome-specific sequences.

Based on these specific sequences, PCR primers were designed using the Primer3Plus online tool^2^ and synthesized by Sangon Biotech (Chengdu, China). The amplified products were electrophoresed in 3% agarose gel. The markers detected in DT23 and *P. huashanica* but absent in CS were identified as 7Ns chromosome-specific molecular markers. The stability, repeatability, and specificity of these markers were validated in CS, CS*ph2b*, *P. huashanica*, and DT23 as well as in 17 wheat-related species. The PCR amplification mixture (in a final volume of 20μl) contained 1μl template DNA (200ng/μL), 10μl of 2× Taq Master Mix for PAGE (Dye Plus), 1.0μl of each primer (10μM), and 7.0μl ddH_2_O. The PCR procedure was as follows: 94°C for 5min, followed by 35cycles of 94°C for 30s, an appropriate annealing temperature of 60–70°C for 30s, and 72°C for 30s, and a final extension at 72°C for 10min.

### FISH Probes Development

The verified molecular markers specific to the Ns genome of *P. huashanica*, *P. juncea*, and *Leymus* species were amplified by PCR from *P. huashanica* genomic DNA. An aliquot (5μl) of the amplicons was electrophoresed in 3% agarose gel to check the product length and yield. The PCR products were extracted with sodium acetate and precipitated with ethanol. The pellets were rinsed with 70% ethanol and dissolved in 30μl ddH_2_O. The extracted DNA fragments were labeled with fluorescein-12-dUTP (Green) using the nick translation method (Thermo Fisher Scientific) and served as probes in the FISH analysis of CS, DT23, *P. huashanica*, and 11 wheat-related species.

## Results

### Chromosomal Constitution of DT23

**Figure 1:**
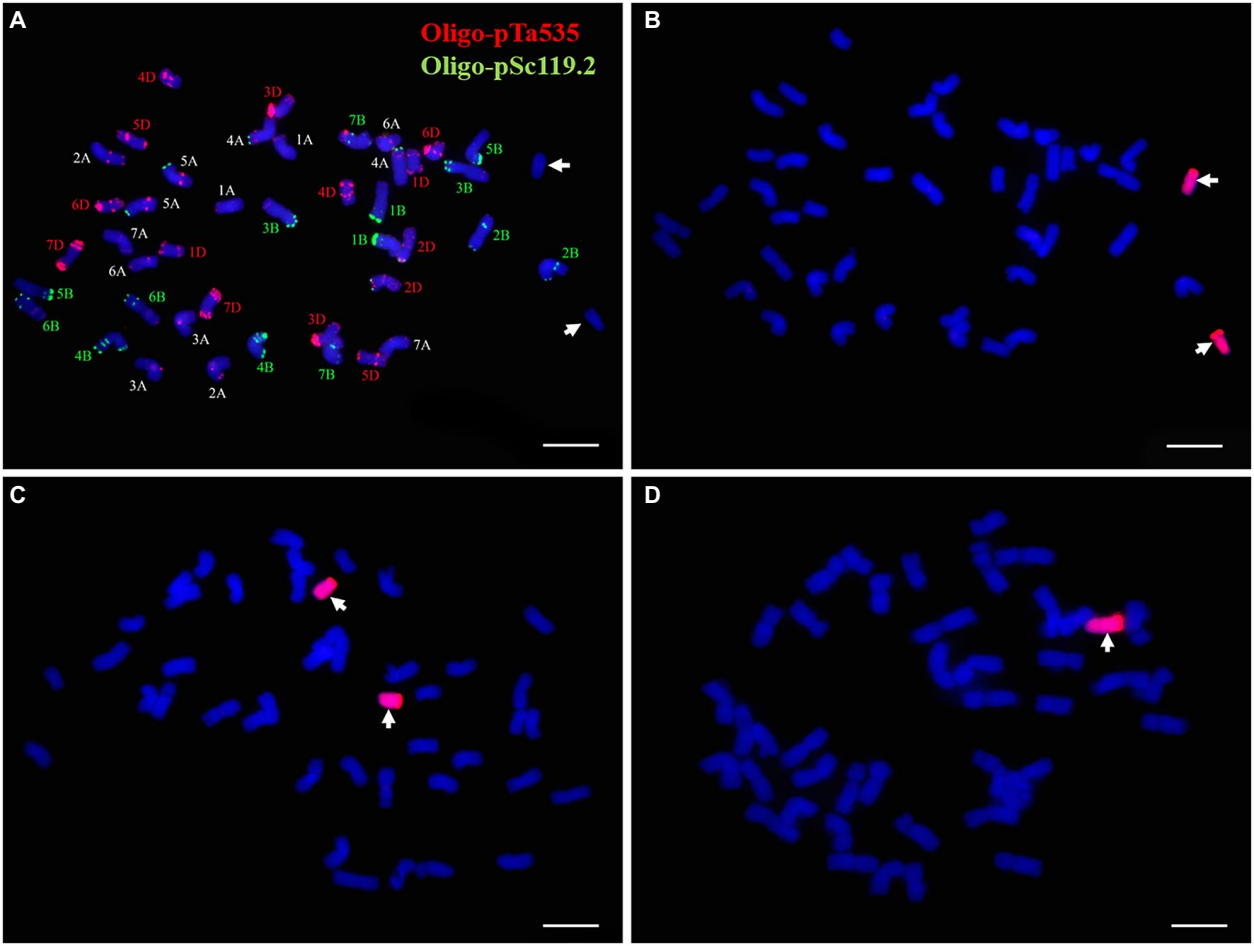
FISH and sequential GISH identification of the wheat-*P. huashanica* ditelosomic addition line DT23. **(A)** FISH identification of DT23 using Oligo-pSc119.2 (green) and Oligo-pTa535(red). **(B)** Sequential GISH analysis on the same metaphase cell of DT23 using *P. huashanica* genomic DNA as a probe (red). **(C)** GISH identification of the selfed progeny of DT23 which carried a pair of Ns telocentric chromosomes. **(D)** GISH identification of the selfed progeny of DT23 which carried one Ns telocentric chromosome. Arrows indicate the introduced *P. huashanica* chromosomes in DT23. Scale bar: 10μm.

FISH and sequential GISH analyses were performed to determine the chromosomal composition of the wheat-*P. huashanica* line DT23. According to the standard FISH karyotype of Chinese Spring (Tang et al., 2014), FISH analysis with the probes Oligo-pSc119.2 (green) and Oligo-pTa535 (red) revealed that DT23 carried 42 wheat chromosomes and two telocentric chromosomes that lacked fluorescent signals (Figure 1A). When *P. huashanica* genomic DNA was used as the probe and J-11 genomic DNA as the blocker, sequential GISH analysis further revealed that the two telocentric chromosomes with strong red hybridization signals were Ns chromosomes from *P. huashanica* (Figure 1B). To confirm the cytological stability of the line DT23, GISH was used to identify 40 randomly selected seeds from selfed progeny of DT23. Thirty-six seeds carried a pair of Ns telocentric chromosomes (Figure 1C), and four seeds carried one Ns telocentric chromosome (Figure 1D). Therefore, these findings suggested that DT23 was a cytogenetically relatively stable wheat-*P. huashanica* ditelosomic addition line.

### FISH Painting Analysis of DT23

**Figure 2:**
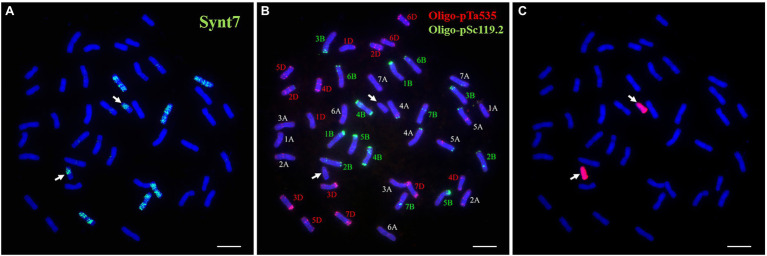
Sequential FISH and GISH analyses of the wheat-*P. huashanica* ditelosomic addition line DT23. **(A)** FISH painting analysis of DT23 using probe Synt7 (green). **(B)** FISH identification of DT23 using Oligo-pSc119.2 (green) and Oligo-pTa535(red). **(C)** *P. huashanica* genomic DNA was used as a probe for GISH (red). Arrows indicate the introduced *P. huashanica* chromosomes in DT23. Scale bar: 10μm.

FISH painting with the probes (Synt1 to Synt7) corresponding to the first to seventh linkage group was performed on DT23. Probe Synt7 painted the six complete chromosomes and two telocentric chromosomes (Figure 2A). Sequential FISH using Oligo-pSc119.2 and Oligo-pTa535 indicated that three pairs of complete chromosomes were painted, comprising the common wheat chromosomes 7A, 7B, and 7D, whereas the two telocentric chromosomes did not show hybridization signals (Figure 2B). GISH analysis revealed that the two telocentric chromosomes with strong red hybridization signals were Ns chromosomes of *P. huashanica* (Figure 2C). Therefore, these results convincingly indicated that the alien chromosomal segments in DT23 belonged to the seventh group and that DT23 is a wheat–*P. huashanica* 7Ns ditelosomic addition line.

### PLUG Marker Analysis of DT23

**Figure 3:**
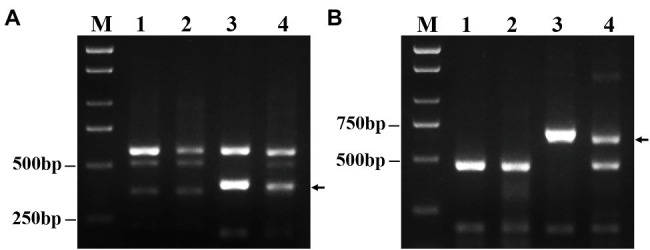
Amplification patterns of wheat PLUG markers. **(A)** *TNAC1782-TaqI*, **(B)** *TNAC1845*-*TaqI*. M: Marker (2000bp); 1: CS; 2: CS*ph2b*; 3: *P. huashanica*; 4: DT23 (wheat-*P. huashanica* ditelosomic addition line). Arrows indicate the diagnostic amplification products for Ns genome.

As expected, among 135 PLUG markers, two markers (*TNAC1782*-7AS 7BS 7DS and *TNAC1845*-7AL 7BL 7DL) distributed on the group 7 chromosomes of wheat amplified the same specific bands in DT23 and *P. huashanica* but not in CS and CS*ph2b* (Figure 3). This result also suggested that *P. huashanica* chromosomal segments introduced into DT23 belonged to the seventh group.

### Observation of Spike Differentiation and Growth Period

During the 2020–2021 growing season, the growth period was investigated in the field for DT23, CS, and CS*ph2b*. Statistical analysis revealed that the timing of the seedling, three-leaf, and tillering stages was consistent among the three materials, but the timing of the jointing to maturity stages in DT23 was strongly accelerated compared with those of CS and CS*ph2b* (Table 2). These findings were identical to those observed under greenhouse conditions (Supplementary Table 1). The entire growth period of DT23 was 14 and 11days shorter than that of CS and CS*ph2b*, respectively (Table 2).

**Table 2:** Growth period statistics of CS, CS*ph2b*, and DT23 in the field.

Materials	Seedling stage	Three-leaf stage	Tillering stage	Jointing stage	Booting stage	Heading stage	Flowering stage	Maturity stage	Whole growth period (days)
CS	November 2, 2020	November 10, 2020	November 18, 2020	February 10, 2021	March 14, 2021	March 23, 2021	March 29, 2021	May 9, 2021	194
CS*ph2b*	November 2, 2020	November 10, 2020	November 18, 2020	February 10, 2021	March 10, 2021	March 18, 2021	March 26, 2021	May 6, 2021	191
DT23	November 2, 2020	November 10, 2020	November 18, 2020	February 4, 2021	February 22, 2021	March 10, 2021	March 19, 2021	April 25, 2021	180

**Figure 4:**
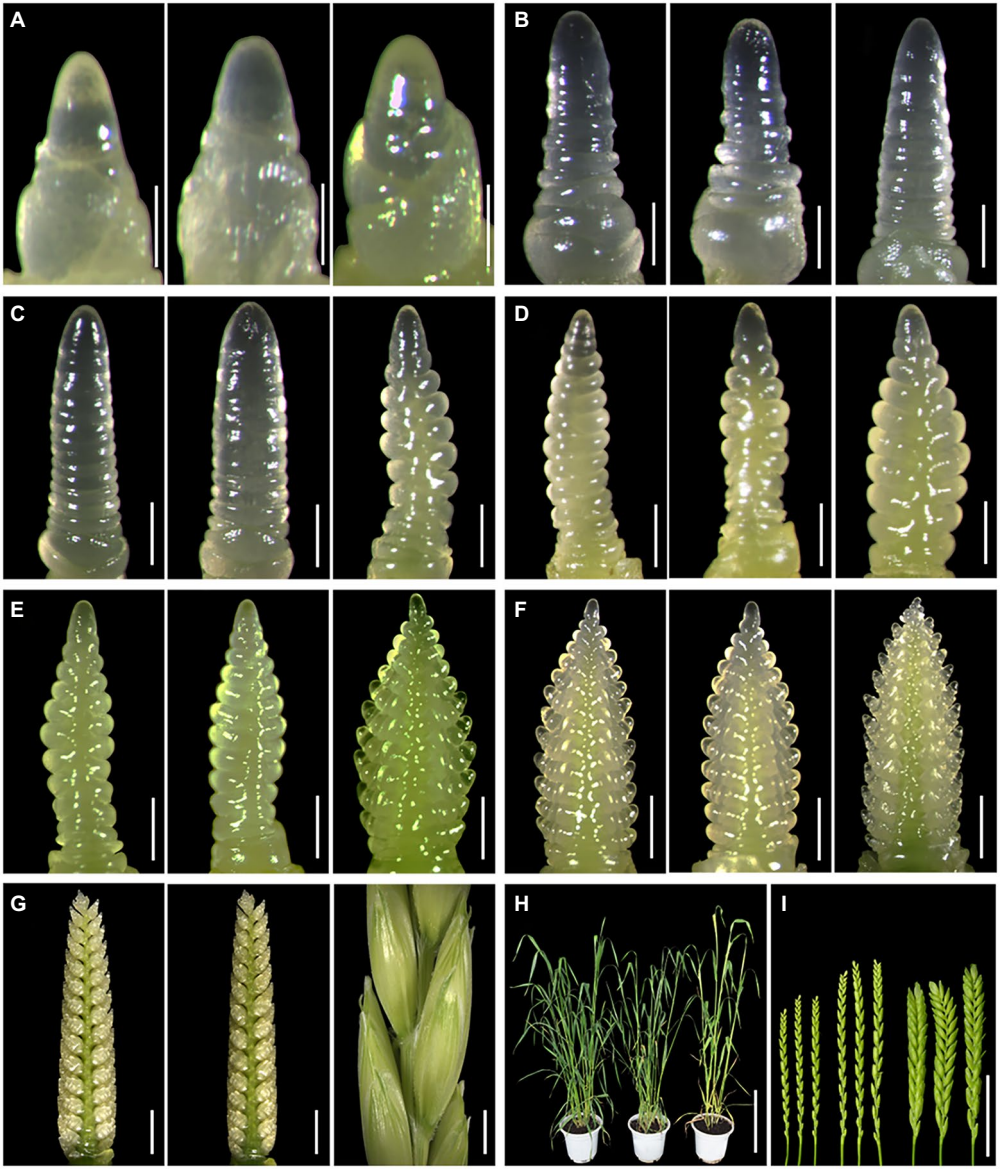
Morphological observation of spike differentiation in different dates under field conditions. Different uppercase letters indicate the date of observation. **(A)** November 17, 2020. **(B)** December 15, 2020. **(C)** December 30, 2020. **(D)** January 12, 2021. **(E)** January 20, 2021. **(F)** February 2, 2021. **(G)** February 24, 2021. **(H,I)** March 10, 2021. Images of each panel from left to right are CS, CS*ph2b* and DT23, respectively. Scale bars: A, 200μm; B-F, 150μm; G, 50μm; H, 2cm; I, 1cm.

To further clarify the phenotypic differences between DT23 and its wheat parents CS and CS*ph2b*, spike differentiation from the three-leaf stage to the heading stage in the field was observed by stereomicroscopy during the 2020–2021 growing season. On November 17, 2020, DT23, CS, and CS*ph2b* plants were all at the apex elongation stage (Table 3, Figure 4A), indicating that no obvious difference in development was observed between DT23 and its wheat parents up to this stage. However, a clear developmental difference was observed on December 15, 2020, when CS and CS*ph2b* plants were at the mid-single-ridge stage, whereas DT23 plants were at the mid-double-ridge stage (Table 3, Figure 4B). Subsequently, DT23 developed more rapidly than CS and CS*ph2b* from the late-double-ridge stage to the tetrad stage (Table 3, Figures 4C–G). When CS and CS*ph2b* were at the booting stage, DT23 plants were already at the heading stage and had developed distinctly larger spikes (Figures 4H,I).

**Table 3:** Spike differentiation of CS, CS*ph2b*, and DT23 at different dates.

Date	CS	CS*ph2b*	DT23
November 17, 2020	Apex elongation stage	Apex elongation stage	Apex elongation stage
December 15, 2020	Middle single-ridge stage	Middle single-ridge stage	Middle double-ridge stage
December 30, 2020	Early double-ridge stage	Early double-ridge stage	Later double-ridge stage
January 12, 2021	Later double-ridge stage	Later double-ridge stage	Glume primordia differentiation stage
January 20, 2021	Glume primordia differentiation stage	Glume primordia differentiation stage	Floret primordia differentiation stage
February 2, 2021	Floret primordia differentiation stage	Floret primordia differentiation stage	Stamen and pistil differentiation stage
February 24, 2021	Anther separation stage	Anther separation stage	Tetrad stage

### Morphology of DT23

**Figure 5:**
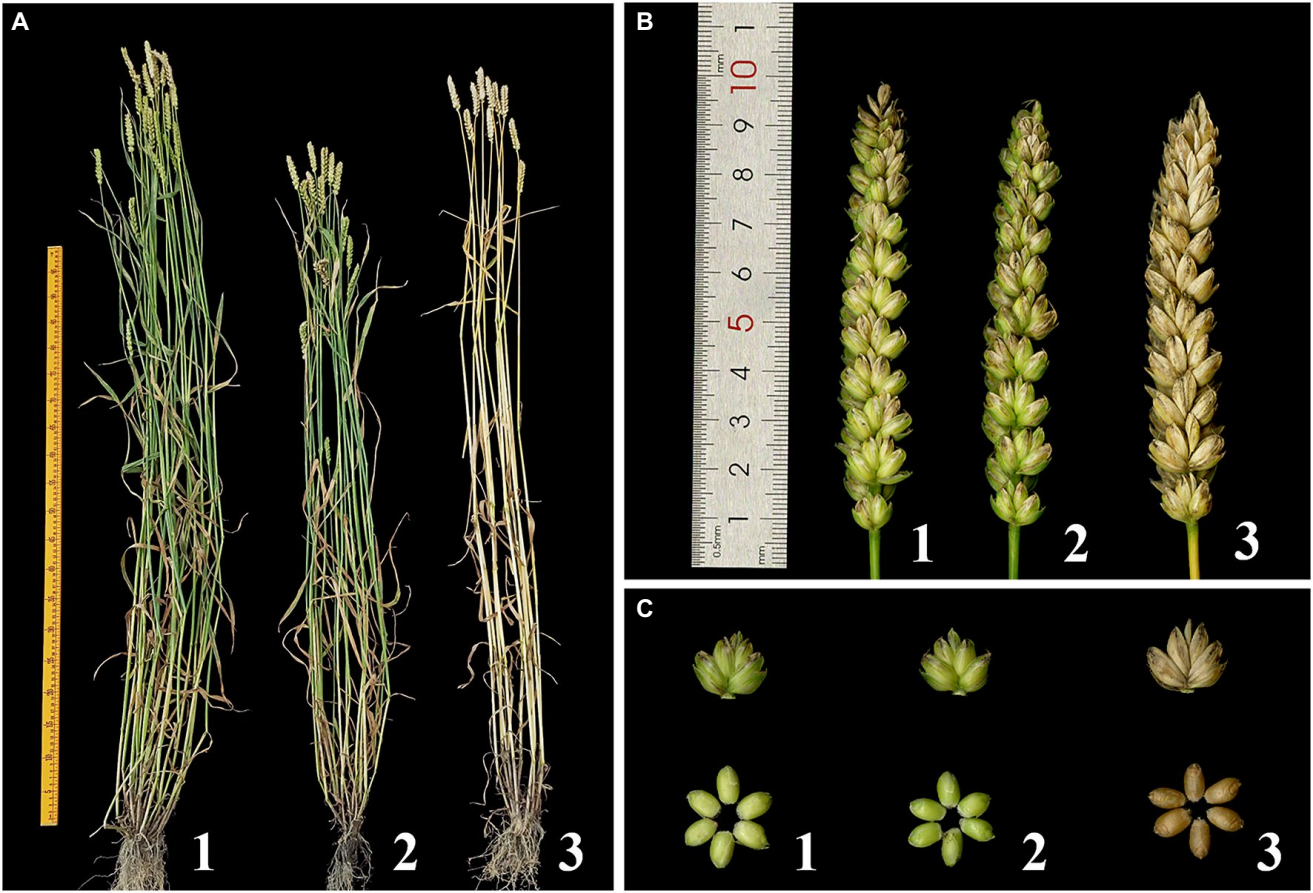
Plant morphology of the wheat-*P. huashanica* ditelosomic addition line DT23 and its wheat parents. **(A)** Adult plants, **(B)** Spikes, and **(C)** Spikelets and grains. 1: CS; 2: CS*ph2b*; 3: DT23.

DT23 showed stability in morphological traits, which were similar to those of the wheat parents CS and CS*ph2b* (Table 4, Figure 5). The average plant height and tiller number of DT23 were significantly lower than those of CS and CS*ph2b*. The 1,000-kernel weight of DT23 was significantly higher than that of CS and CS*ph2b*. No significant differences between DT23 and either CS or CS*ph2b* were observed with regard to spike length, number of spikelets per spike, and number of kernels per spike.

**Table 4:** Agronomic traits of DT23 and its wheat parents.

Lines	Plant height (cm)	Tiller number	Spike length (cm)	Spikelets per spike	Kernels per spike	1,000-kernel weight (g)
CS	145.5 ± 1.0 Aa	19.3 ± 1.1 Aa	9.91 ± 0.11 Aa	23.60 ± 0.33 Aa	55.2 ± 2.3 Aa	29.94 ± 0.28 Aa
CS*ph2b*	139.9 ± 1.4 Bb	14.8 ± 1.0 Bb	9.76 ± 0.13 Aa	23.15 ± 0.31 Aa	57.7 ± 1.4 Aa	29.67 ± 0.32 Aa
DT23	127.5 ± 0.7 Cc	8.35 ± 0.5 Cc	9.84 ± 0.13 Aa	22.95 ± 0.30 Aa	53.9 ± 1.4 Aa	36.80 ± 0.27 Bb

*Data in the columns indicate means±standard errors. Different uppercase and lowercase letters following the means indicate significant differences at the p<0.01 and p<0.05 levels, respectively*.

### Molecular Marker Development

A total of 6,963,342, 3,723,959, and 16,237,871 reads were, respectively, obtained for CS*ph2b*, *P. huashanica* ZY3156, and DT23 using the SLAF-seq approach. The average Q30 score was 94.89%, and the GC content was 48.56%. After filtering out low-quality, repeat, and ambiguous reads, a total of 399,489, 120,418, and 462,445 effective SLAFs were generated for CS*ph2b*, *P. huashanica*, and DT23, respectively. The average sequencing depth was 13.95×. These results were optimal and fulfilled the expected requirements. Sequence comparison revealed that 54 DT23 sequences showed 0% homology with the CS reference genome and less than 23% homology with CS*ph2b* sequences, but more than 90% homology with *P. huashanica* sequences. These sequences were considered to be candidates for specific sequences of chromosome 7Ns from *P. huashanica*.

**Figure 6:**
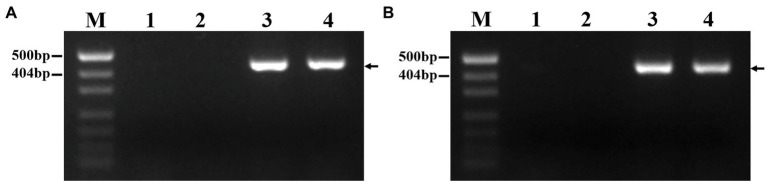
PCR amplification of specific molecular markers. **(A)** PH7Ns-12, **(B)** PH7Ns-38. M: Marker (500bp); 1: CS; 2: CS*ph2b*; 3: *P. huashanica*; 4: DT23 (wheat-*P. huashanica* ditelosomic addition line). Arrows show the diagnostic amplification products of *P. huashanica* 7Ns chromosome.

To develop *P. huashanica* 7Ns chromosome-specific molecular markers, 52 primer pairs were designed based on the candidate specific sequences and used to amplify sequences from CS, CS*ph2b*, *P. huashanica*, and DT23 (Supplementary Table 2). In total, 45 primer pairs amplified specific bands from DT23 and *P. huashanica* but not from CS and CS*ph2b*, such as PH7Ns-12 and PH7Ns-38 (Figures 6A,B). Therefore, these markers were regarded as *P. huashanica* 7Ns chromosome-specific molecular markers, with a success rate of up to 86.54%.

**Figure 7:**
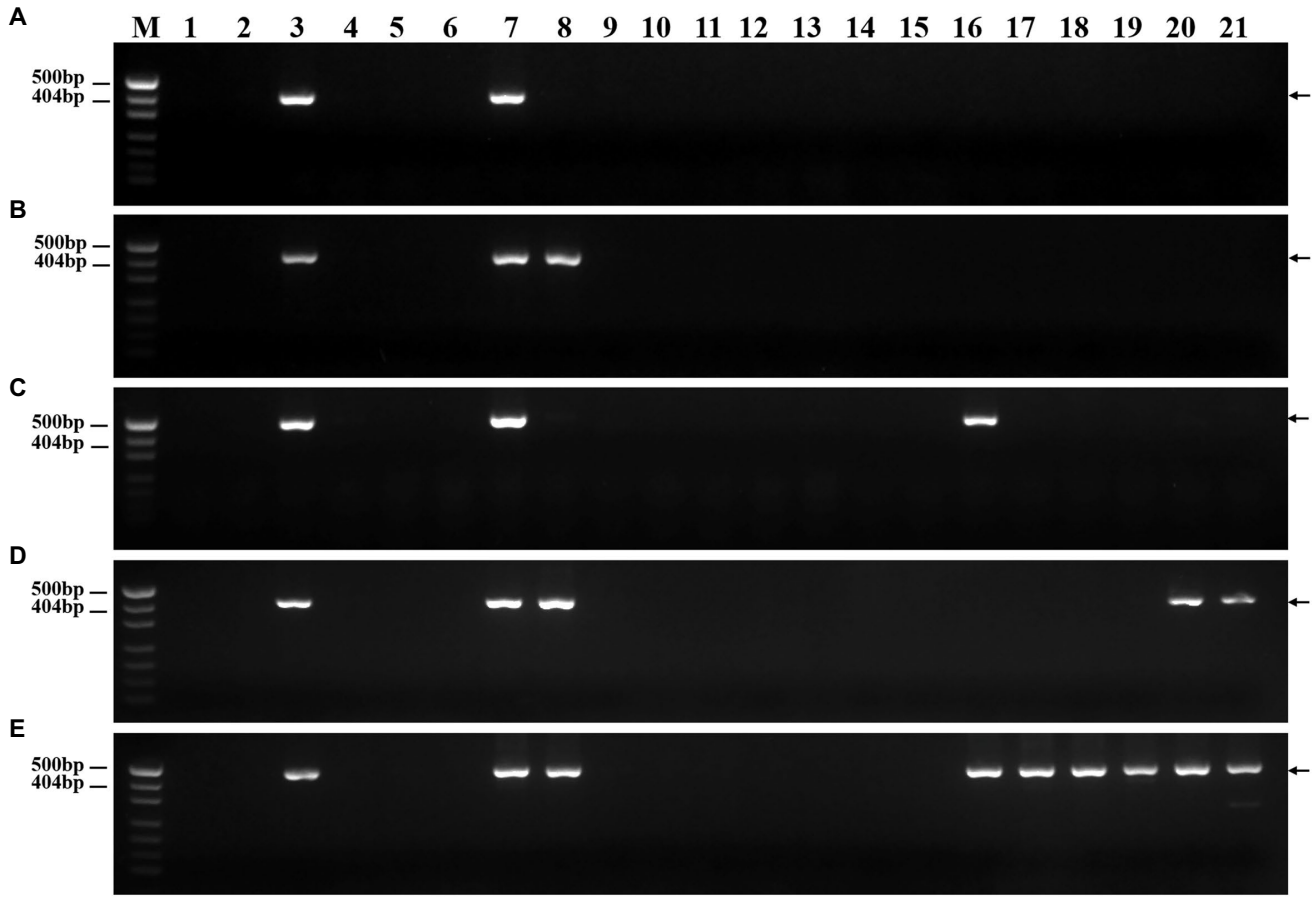
Specificity and stability of specific molecular markers in other wheat-related species. (A) PH7Ns-15, (B) PH7Ns-43, (C) PH7Ns-12, (D) PH7Ns-14, and (E) PH7Ns-5. M: Marker (500bp); 1: CS; 2: CS*ph2b*; 3: DT23 (wheat-*P. huashanica* ditelosomic addition line); 4: *T. urartu*; 5: *Ae. speltoides*; 6: *Ae. tauschii*; 7: *P. huashanica*; 8: *P. juncea*; 9: *S. cereale*; 10: *H. vulgre*; 11: *Ag. cristatum*; 12: *Das. villosum*; 13: *Pse. libanotica*; 14: *Th. elongatum*; 15: *Th. bessarabicum*; 16: *L. racemosus*; 17: *L. secalinus*; 18: *L. coreanus*; 19: *L. multicaulis*; 20: *L. arenarius*; and 21: *L. cinereus*. Arrows show the diagnostic amplification products of *P. huashanica* 7Ns chromosome.

To verify their specificity and stability, the 45 molecular markers were used to amplify sequences from 17 wheat-related species. The PCR amplification results are presented in Supplementary Table 3. Among these markers, two markers amplified sequences only from *P. huashanica* but not from the wheat-related species (Figure 7A). In contrast, one and two markers amplified specific sequences not only from *P. huashanica*, but also from *L. racemosus* and *P. juncea*, respectively (Figures 7B,C). In addition, one marker amplified a sequence common to *P. huashanica*, *P. juncea*, *L. arenarius*, and *L. cinereus*, but not from any other wheat-related species (Figure 7D). Thirteen markers amplified specific sequences not only from *P. huashanica* and *P. juncea*, but also from six *Leymus* species (Figure 7E). Furthermore, specific bands were amplified for the other wheat-related species. Five, 3, 4, 5, 6, 9, 5, 11, 15, and 4 markers amplified specific sequences from *T. urartu, Aegilops speltoides*, *Ae. tauschii, Secale cereale*, *H. vulgare*, *Agropyron cristatum*, *Dasypyrum villosum*, *Th. bessarabicum*, *Thinopyrum elongatum*, and *Pseudoroegneria libanotica*, respectively.

### FISH Probes Development

**Figure 8:**
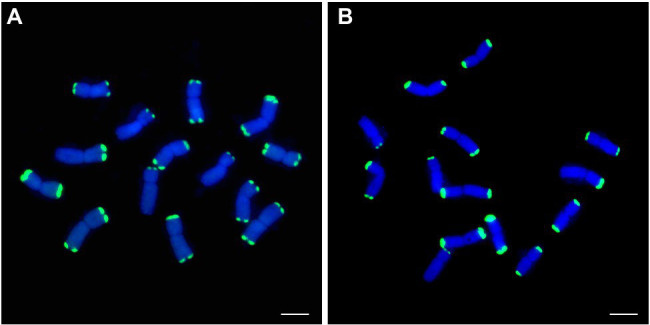
FISH patterns of repetitive DNA probes in *P. huashancia*. **(A)** pPh15 (green), **(B)** pPh37 (green). Scale bar: 10μm.

To develop *P. huashanica*-specific FISH probes, PCR products of 19 markers, comprising two markers specific to *P. huashanica*, two markers specific to *P. huashanica* and *P. juncea*, and 15 markers specific to the Ns genome-containing species, were fluorescently labeled as probes for FISH analysis of *P. huashanica*. A total of 19 *P. huashanica*-specific FISH probes were developed (Supplementary Table 4). All probes produced strong and distinct hybridization signals in telomeric regions of every chromosome for *P. huashanica* (Supplementary Figure 1), comprising 10 chromosomes with two telomeric signals and four chromosomes with one telomeric signal, such as pPh15 and pPh37 (Figures 8A,B).

**Figure 9:**
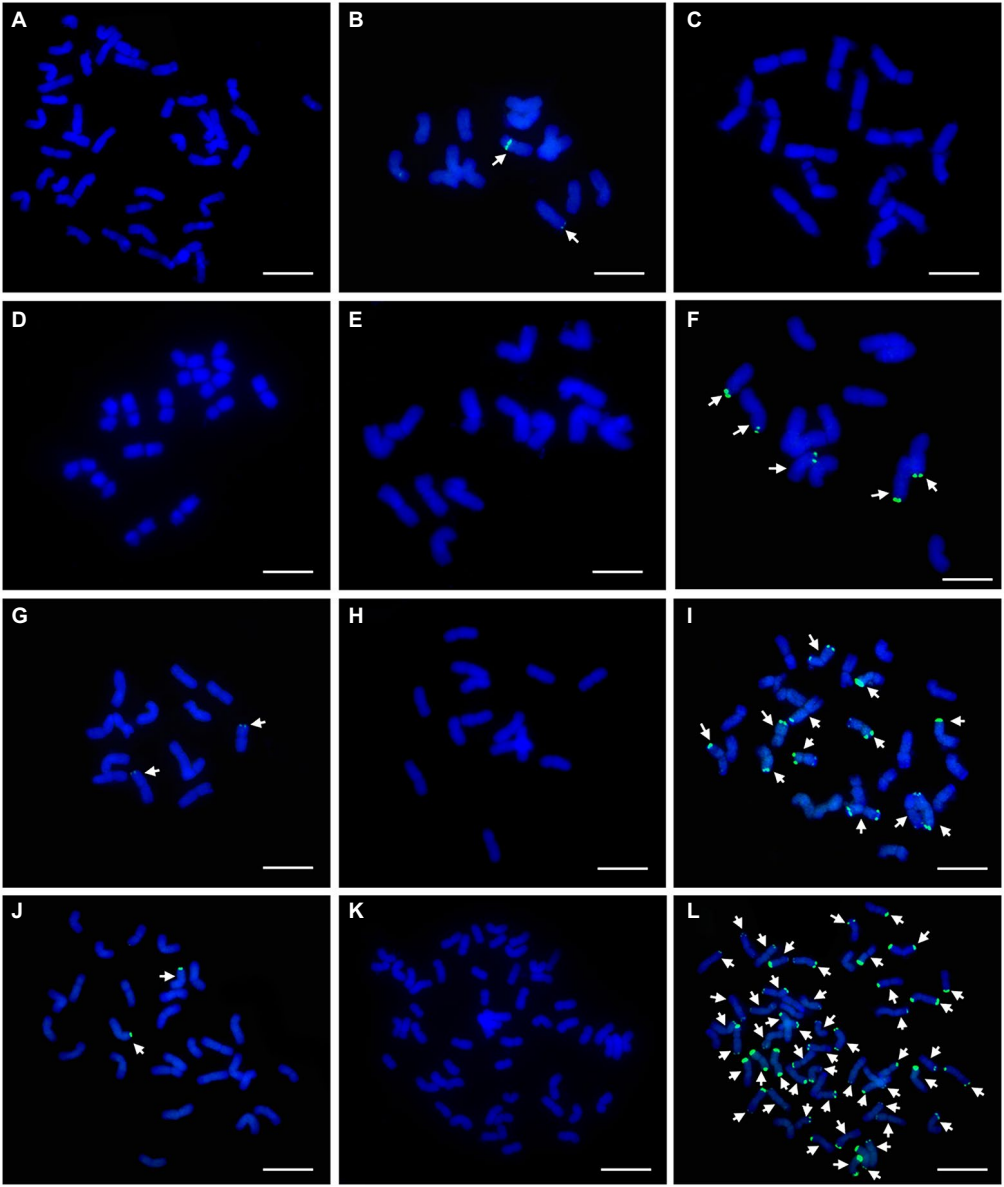
FISH patterns of repetitive DNA probe pPh37 (green) in CS and 11 wheat-related species. **(A)** CS; **(B)** *P. juncea*; **(C)** *S. cereal*; **(D)** *H. vulgre*; **(E)** *Ag. cristatum*; **(F)** *Das. villosum*; **(G)** *Pse. libanotica*; **(H)** *Th. elongatum*; **(I)** *L. racemosus*; **(J)** *L. multicaulis*; **(K)** *L. arenarius*; and **(L)** *L. cinereus*. Arrows indicate partial chromosomes with strong hybridization signals. Scale bar: 10μm.

To assess the utility of these probes, further FISH analysis with probe pPh37 was performed on CS and 11 wheat-related species. *P. juncea* carried two chromosomes with distinct hybridization signals in telomeric and middle chromosomal arm regions (Figure 9B), *Das. villosum*, *Pse. libanotica*, and *L. multicaulis* carried five, two, and two chromosomes, respectively, with strong hybridization signals in telomeric regions (Figures 9F,G,J). In addition, *L. racemosus* and *L. cinereus* had 12 and 50 chromosomes, respectively, with strong hybridization signals in telomeric and subtelomeric regions (Figures 9I,L). No hybridization signals were observed on chromosomes of CS, *S. cereale*, *H. vulgare*, *Ag. cristatum*, *Th. elongatum*, and *L. arenarius* (Figures 9A,C–E,H,K).

**Figure 10:**
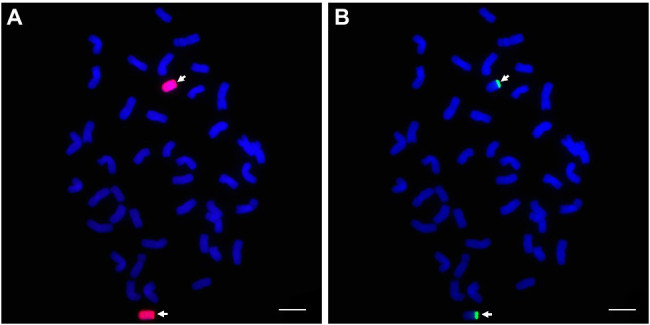
GISH and sequential FISH identification of the wheat-*P. huashanica* ditelosomic addition line DT23. **(A)** *P. huashanica* genomic DNA was used as a probe for GISH (red). **(B)** FISH identification of DT23 using pPh37 (green). Arrows indicate the introduced *P. huashanica* chromosomes in DT23. Scale bar: 10μm.

In comparison with the GISH pattern of *P. huashanica* genomic DNA as a probe (Figure 10A), the probe pPh37 generated strong hybridization signals in telomeric regions of the two alien chromosomal segments in line DT23, but not on common wheat A-, B-, and D-genome chromosomes (Figure 10B). This result indicated that the probes developed in this study could be applied to detect *P. huashanica* chromosomes or chromosomal segments in a wheat background.

## Discussion

Wild relatives of wheat are important for the breeding of new early maturing genotypes and to broaden the genetic base in wheat breeding programs. Early maturing germplasm has previously been generated by crossing common wheat with wild relatives. For instance, Efremova et al. (1996) developed 12 alien 5R(5A) disomic substitution lines with different wheat genetic backgrounds using the alien substitution line “Saratovskaya 29” 5R(5A) as a donor. Efremova et al. (2006) demonstrated that these lines differed in timing of ear emergence and response to vernalization. Bao et al. (2009) developed a new dwarf and early maturing germplasm line Shannong 0057–2 derived from the BC_3_F_6_ progeny of the cross between common wheat “Yannong 15” and *Th. intermedium*. Liu et al. (2011) produced two early maturing disomic addition lines, WB0528 and WB0647, selected from the hybrid progenies between common wheat and cultivated barley. Farkas et al. (2013) suggested that the addition of different barley chromosomes significantly influenced the flowering time of wheat both in controlled environment tests and in the field. The authors reported that the wheat-barley 7H addition line was consistently the earliest flowering, whereas the wheat-barley 4H addition line was the latest to flower in all treatments. Wang et al. (2015) identified a novel wheat-*P. huashanica* 6Ns disomic addition line that exhibited earlier maturation than its wheat parent and suggested that *P. huashanica* 6Ns chromosomes significantly affected the spike primordium development and flowering time of wheat in the field. Wang et al. (2017) developed a new germplasm line with early maturation derived from hybridization between common wheat “Yannong 15” and *Ae. ventricosa* × *Ae. cylindrica* amphiploid SDAU18. However, few early maturing germplasm lines derived from wide hybrids between common wheat and *P. huashanica* have been reported previously. In the present study, we developed and characterized a new wheat-*P. huashanica* 7Ns ditelosomic addition line DT23 derived from the *P. huashanica*/CS*ph2b*/CS F_4_ progeny. The maturity stage of DT23 was 14 and 11days earlier than that of the parents CS and CS*ph2b*, respectively. Morphological observation revealed that spike differentiation of DT23 and the parents CS and CS*ph2b* displayed distinct differences after the apex elongation stage. The main reason for the differences was that spike differentiation of DT23 was more rapid than that of CS and CS*ph2b* during the single-ridge to double-ridge stages, thereby giving rise to the subsequent discrepancy in timing of maturity. The pedigree provided the only evidence that DT23 carried novel *Eps* gene(s) from the *P. huashanica* 7Ns chromosome. All seven Ns chromosomes of *P. huashanica* have been verified to be useful on account of the vast number of beneficial genes carried: Stripe rust resistance genes are located on 1Ns, 2Ns, 3Ns, 4Ns, and 5Ns (Du et al., 2014a,c,d; Li et al., 2019; Qu et al., 2021); leaf rust resistance genes are located on 1Ns and 7Ns (Du et al., 2013c, 2014b); powdery mildew resistance genes are located on 1Ns, 3Ns, 4Ns, and 5Ns (Han et al., 2020 Li et al., 2020a 2021a Liu et al., 2021); a take-all resistance gene is located on 2Ns (Bai et al., 2020); gluten and gliadin-related genes are located on 1Ns and 6Ns (Du et al., 2013b; Qu et al., 2021); and early maturity-related genes are located on 6Ns (Wang et al., 2015). The present study is the first report of new *Eps* gene(s) probably associated with a group 7 chromosome of *P. huashanica*. Furthermore, the additional chromosomal segments have no obvious genetic linkage drag affecting agronomic performance. Accordingly, the ditelosomic addition line DT23, which exhibits an early maturation phenotype, may represent valuable germplasm for breeding early maturity wheat cultivars.

Molecular markers for detecting and tracking alien chromosomes and/or chromosomal segments carrying elite genes are of vital importance in wheat breeding programs (Fedak, 1999). Conventional methods previously used to develop markers for *P. huashanica* are time-consuming, imprecise, and expensive, such as random-amplified polymorphic DNA (RAPD) and sequence-characterized amplified region (SCAR) markers. For example, Chen et al. (2010) screened three *P. huashanica* genome-specific repetitive sequences using 200 RAPD primers. Subsequently, five Ns-specific and 11 5Ns-specific SCAR markers for *P. huashanica* were developed (Wang et al., 2013; Du et al., 2013d; Wang et al., 2014a,b; Zhang et al., 2017; Li et al., 2020b). The SLAF-seq approach, a high-throughput, high accuracy, low cost, and next-generation sequencing-based technology, has been applied successfully to develop large numbers of highly accurate molecular markers in a variety of wild relatives of wheat, such as *Th. elongatum* (Chen et al., 2013), *Th. intermedium* (Li et al., 2016), *Th. ponticum* (Yang et al., 2021), *S. cereale* (Du et al., 2018), and *Ae. biuncialis* (Song et al., 2020). To date, no *P. huashanica*-specific SLAF-based markers have been reported. In the current study, we developed 45 specific markers for chromosome 7Ns of *P. huashanica* in the ditelosomic addition line DT23 based on SLAF-seq data, with a success rate of up to 86.54%. These markers may be potentially useful not only for tracking *P. huashanica* 7Ns chromosomal segments harboring *Eps* gene(s) in a wheat background, but also for distinguishing the Ns genome of *P. huashanica* and other closely related genomes from Triticeae species. In addition, marker validation analyses indicated that the marker amplification frequencies in *P. juncea* (Ns) and *Leymus* species (NsXm) were much higher than that in other wheat-related species. These findings suggested that the relationship between *Psathyrostachys* and *Leymus* is closer than that with other wild relatives, which supported evidence that the Ns genome of *Leymus* was donated by *Psathyrostachys* (Zhang and Dvořák, 1991; Sha et al., 2017).

FISH analysis with repetitive DNA probes has been widely used to identify alien chromosomes and/or chromosomal segments integrated into a common wheat background (Cseh et al., 2011; González-García et al., 2011). For instance, Li et al. (2016) produced a novel FISH probe pSt122 representing terminal repeats from *Th. intermedium* using SLAF-seq. Liu et al. (2018a) generated a blue grain-related FISH probe pThp12.19 from the wheat-*Th. ponticum* 4Ag (4D) disomic substitution line Blue 58 by SLAF-seq. Liu et al. (2018b) developed eight *Th. ponticum*-specific FISH probes based on SLAF-seq. In the present study, we developed 19 *P. huashanica*-specific FISH probes by SLAF-seq, which produced strong and identical fluorescent signals in one or both telomeric regions of all *P. huashanica* chromosomes, but not in wheat chromosomes. Compared with GISH using genomic DNA of *P. huashanica* as a probe, FISH analysis with these probes was successfully applied to detect *P. huashanica* chromosomal segments in DT23. To the best of our knowledge, this is the first report of *P. huashanica*-specific FISH probes. Furthermore, the probes provide the possibility of distinguishing different *P. huashanica* chromosomes together with other FISH probes. Therefore, these probes will be convenient and applicable for discrimination of *P. huashanica* chromosomes and/or chromosomal segments in a wheat background and for identification of chromosomes from other wheat-related species. The present specificity analysis suggested that probe pPh37 generated strong hybridization signals from 12 *L. racemosus* chromosomes and two *L. multicaulis* chromosomes, but not *L. arenarius* chromosomes. These findings revealed that the donor species of the Ns genome to *Leymus* was not *P. huashanica*, which was consistent with the inferences of Bödvarsdóttir and Anamthawat-Jónsson (2003) and Wang et al. (2006) based on DNA hybridization and FISH patterns.

## Conclusion

Herein, a novel wheat-*P. huashanica* 7Ns ditelosomic addition line, DT23, was identified by FISH, GISH, PLUG marker, and FISH painting analyses. Compared with the wheat parents, DT23 exhibits earlier maturation. Hence, it can be employed as a valuable intermediate material for breeding early maturing wheat cultivars. In addition, 45 *P. huashanica* 7Ns chromosome-specific markers and 19 *P. huashanica*-specific FISH probes were developed based on SLAF-seq. The newly developed markers and probes will be useful for accurate detection of *P. huashanica* chromosomes and/or chromosomal segments in a wheat background as well as chromosomes from other closely related species.

## Data Availability Statement

The original contributions presented in the study are included in the article/S**upplementary Material**, further inquiries can be directed to the corresponding author.

## Author Contributions

BT, LZ, LL, and HK conducted the experiment, analyzed the data, and drafted the manuscript. HZ, WZ, LX, YW, and JZ developed addition line and evaluated morphological traits. XF, LS, HZ, DW, YC, and GC provided technique guidance. YZ and HK designed the experiment and formulated the questions. All authors contributed to the article and approved the submitted version.

## Funding

This work was supported by the National Natural Science Foundation of China (Nos. 31771781 and 31971883), and the Applied Basic Research Programs of the Science and Technology Bureau of Sichuan Province (2020YJ0348), and the Science and Technology Bureau of Chengdu City (2021-YF05-00681-SN).

## Conflict of Interest

The authors declare that the research was conducted in the absence of any commercial or financial relationships that could be construed as a potential conflict of interest.

## Publisher’s Note

All claims expressed in this article are solely those of the authors and do not necessarily represent those of their affiliated organizations, or those of the publisher, the editors and the reviewers. Any product that may be evaluated in this article, or claim that may be made by its manufacturer, is not guaranteed or endorsed by the publisher.

## Footnotes

^1^ https://urgi.versailles.inra.fr/download/iwgsc/IWGSC_RefSeq_Assemblies/v1.0/

^2^ http://www.primer3plus.com/cgi-bin/dev/primer3plus.cgi

## Supplementary Material

Supplementary Table 1 Growth period statistics of CS, CS*ph2b*, and DT23 in the greenhouse.

Supplementary Table 2 PCR amplification results of *P. huashanica* molecular markers.

Supplementary Table 3 Specific amplification of 7Ns chromosome markers in wheat-related species.

Supplementary Table 4 SLAF sequences of the *P. huashanica*-specific FISH probes.

Supplementary Figure 1 FISH patterns of 19 repetitive DNA probes in *P. huashancia*. **(A)** pPh1, **(B)** pPh3, **(C)** pPh5, **(D)** pPh12, **(E)** pPh13, **(F)** pPh14, **(G)** pPh15, **(H)** pPh18, **(I)** pPh19, **(J)** pPh24, **(K)** pPh27, **(L)** pPh32, **(M)** pPh33, **(N)** pPh37, **(O)** pPh38, **(P)** pPh43, **(Q)** pPh48, **(R)** pPh49, and **(S)** pPh50. All probes are green. Scale bar: 10μm.
