# Supplementary figures and images for: Identification of a Wheat-Psathyrostachys huashanica 7Ns Ditelosomic Addition Line Conferring Early Maturation by Cytological Analysis and Newly Developed Molecular and FISH Markers
Source: Front Plant Sci. 2021 Dec 9;12:784001. doi: 10.3389/fpls.2021.784001 (PMC8695443; doi:10.3389/fpls.2021.784001)

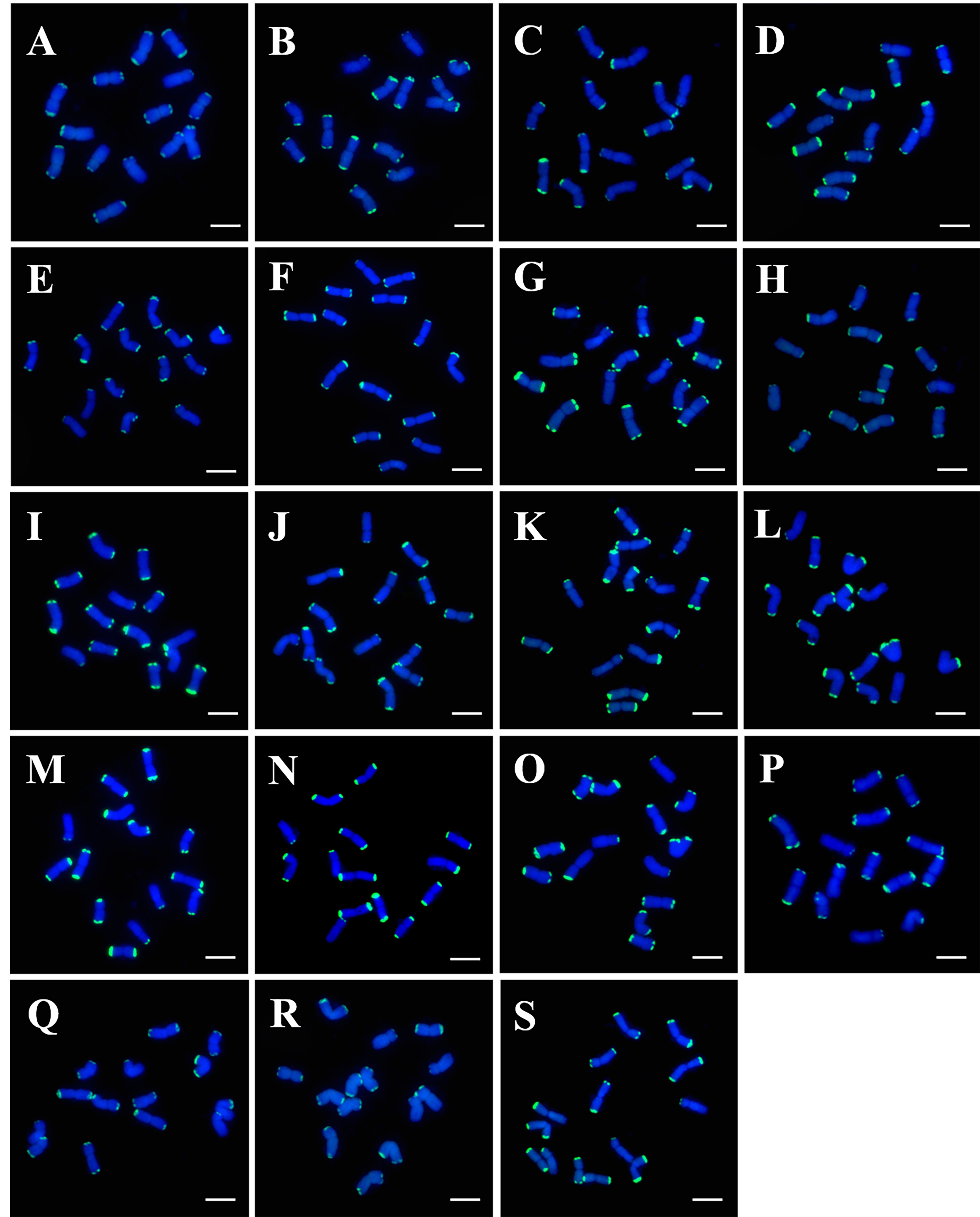

Supplement: Supplementary Figure 1 — FISH patterns of 19 repetitive DNA probes in P. huashancia. (A) pPh1, (B) pPh3, (C) pPh5, (D) pPh12, (E) pPh13, (F) pPh14, (G) pPh15, (H) pPh18, (I) pPh19, (J) pPh24, (K) pPh27, (L) pPh32, (M) pPh33, (N) pPh37, (O) pPh38, (P) pPh43, (Q) pPh48, (R) pPh49, and (S) pPh50. All probes are green. Scale bar: 10μm. [file Image_1.TIF]
